# MiR‐223‐3p alleviates trigeminal neuropathic pain in the male mouse by targeting MKNK2 and MAPK/ERK signaling

**DOI:** 10.1002/brb3.2634

**Published:** 2022-05-24

**Authors:** Bixia Huang, Shaoyong Guo, Yipan Zhang, Pengxing Lin, Changgui Lin, Meixia Chen, Shengyin Zhu, Liyu Huang, Junwei He, Lingfeng Zhang, Yanping Zheng, Zhipeng Wen

**Affiliations:** ^1^ Department of Neurology The Affiliated Hospital of Putian University Putian China; ^2^ Department of Stomatology The First Hospital of Putian City Putian China

**Keywords:** MAPK/ERK signaling, miR‐223‐3p, MKNK2, trigeminal neuralgia

## Abstract

**Background:**

Trigeminal neuralgia (TN) is a neuropathic pain that occurs in branches of the trigeminal nerve. MicroRNAs (miRNAs) have been considered key mediators of neuropathic pain. This study was aimed to elucidate the pathophysiological function and mechanisms of miR‐223‐3p in mouse models of TN.

**Methods:**

Infraorbital nerve chronic constriction injury (CCI‐ION) was applied in male C57BL/6J mice to establish mouse models of TN. Pain responses were assessed utilizing Von Frey method. The expression of miR‐223‐3p, MKNK2, and MAPK/ERK pathway protein in trigeminal ganglions (TGs) of CCI‐ION mice was measured using RT‐qPCR and Western blotting. The concentrations of inflammatory cytokines were evaluated using Western blotting. The relationship between miR‐223‐3p and MKNK2 was tested by a luciferase reporter assay.

**Results:**

We found that miR‐223‐3p was downregulated, while MKNK2 was upregulated in TGs of CCI‐ION mice. MiR‐223‐3p overexpression by an intracerebroventricular injection of Lv‐miR‐223‐3p attenuated trigeminal neuropathic pain in CCI‐ION mice, as well as reduced the protein levels of pro‐inflammatory cytokines in TGs of CCI‐ION mice. MKNK2 was verified to be targeted by miR‐223‐3p. Additionally, miR‐223‐3p overexpression decreased the phosphorylation levels of ERK1/2, JNK, and p38 protein in TGs of CCI‐ION mice to inhibit MAPK/ERK signaling.

**Conclusions:**

Overall, miR‐223‐3p attenuates the development of TN by targeting MKNK2 to suppress MAPK/ERK signaling.

## Graphical abstract

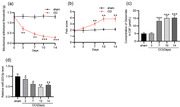

## 1 INTRODUCTION

Craniofacial neuropathic pain is related to various pathologies, such as trigeminal neuralgia (TN), viral or tumoral neuropathies, and nerve entrapments, which usually lead to debilitating chronic painful disorders that are hard to treat (García‐Magro et al., 2021). TN is an excruciating facial pain, accompanied by recurrent neuropathic pain and allodynia in the trigeminal area (Haviv et al., 2016; Zakrzewska & McMillan, 2011). The pain of TN is usually described as an electric shock, shooting, sharp, burning, and lancinating sensation (Yang et al., 2016). Its etiology is involved in injury of the nerve structure induced by trauma, neurovascular compression, or demyelinating disease (Leclercq et al., 2013). Evidence shows that neuroinflammation that could be triggered by factors such as infection, and trauma is a common accompaniment in the development of neuropathic pain (Deng et al., 2017; Shibuta et al., 2012). Anticonvulsant drugs‐dominated pharmacological therapy has been the main treatment for TN. Unfortunately, significant side effects associated with these treatments are life‐threatening during acute and chronic therapy (K.W. Li et al., 2014). Therefore, exploring the processes and mechanisms related to neuropathic facial pain may contributor to better treatment of TN.

MicroRNAs (miRNAs) are short single‐stranded RNAs of about 22 nt in length and act as key regulators of biological processes by controlling gene expression via targeting mRNA 3′‐UTR (Bartel, 2009). They significantly regulate both immune and neuronal processes, therefore, representing prospective targets as possible treatment strategies for neuropathic pain (Kress et al., 2013). Amounting studies show that alterations in the expression levels of numerous miRNAs in pain are crucial for initiating and maintaining neuropathic pain (Andersen et al., 2014). As reported, a study indicated that expression levels of miR‐146b‐5p, −384, 155‐5p, and −132‐3p are markedly higher in the serum of TN patients than in healthy controls (X. Li et al., 2020). MiR‑195 is upregulated in the caudal brain stem of TN rat models and its silence alleviates trigeminal neuropathic pain through inhibiting Sonic Hedgehog signaling activation (Wang et al., 2019). MiR‐186 suppresses neuroinflammation and neuropathic pain in prosopalgia mice by negatively controlling the NLRP3 inflammasome signaling (Chen et al., 2018). Thus, investigation of miRNAs is seemingly a promising tool in elucidating molecular mechanisms of TN development. Interestingly, a study showed that miR‐223 is markedly inhibited in trigeminal ganglions (TGs) from trigeminal neuropathic pain mice (Chen et al., 2018). Moreover, electroacupuncture alleviates postherpetic neuralgia by upregulating miR‐223‐3p expression in spinal cord tissues of rats (Zou et al., 2021); miR‐223‐3p might be a key miRNA in the relief of neuralgia by electroacupuncture (Zou et al., 2019). These studies suggest that miR‐223‐3p could serve as a significant player in the pathogenesis of neuralgia. Thus, targeting miR‐223‐3p may be a promising approach for improving pain relief in pain conditions.

Here, the analgesic effect of miR‐223‐3p was assessed through intracerebroventricular injection of miR‐223‐3p in mouse models of TN. Additionally, a downstream pathway addressed by miR‐223‐3p was investigated in TN to describe the mechanisms how miR‐223‐3p regulates neuropathic pain.

## 2 MATERIALS AND METHODS

### 2.1 Animals

Animal experiments were approved by the Animal Care Committee at Hubei Province Center for Disease Control and Prevention (approval number: 202110021) and were conducted according to its guidelines. Eighty male C57BL/6J mice weighing 2 0 ± 2 g were provided by Vital River Co. Ltd (Beijing, China) and were housed four to five per cage under a pathogen‐free condition (humidity: 55 ± 5%; temperature: 23 ± 2°C) with a 12/12‐h light–dark cycle. Food and water were available ad libitum. Animals were given 1 week of acclimation before experiments, and they were matched by body weight and age for each group.

### 2.2 Infraorbital nerve chronic constriction injury model

An infraorbital nerve chronic constriction injury (CCI‐ION) model was established following the procedures established previously (Guo et al., 2018). C57BL/6J mice were intraperitoneally injected with 3.5% chloral hydrate (1 ml/100 g) for anesthesia, and then received the surgery. Hair from the surgical area was removed, and the skin was disinfected. Under the surgical microscope, a curved incision was made on the superciliary arch to expose the frontal and nasal bones and the eye sockets. The orbital contents were removed along the superior orbital margin with a nerve dissection to expose the infraorbital nerve located on the medial side of the fundus. The infraorbital nerve was dissected free by making a 1‐cm‐long incision in the gingivobuccal margin and were ligated with two chromium wires (5‐0) loosely, separated by 2 mm. The diameter of the infraorbital nerve was slightly reduced but without blocking nerve conduction. For the sham group, the infraorbital nerve was separated without ligation. The incision was sutured with 5‐0 silk sutures. After the animals were awake, they were given antibiotics to prevent infection.

### 2.3 Intracerebroventricular catheter insertion

After anesthesia, the head of mice was fixed on a stereotaxic frame, and the right lateral ventricle was implanted with a 21‐gauge stainless‐steel guide cannula. The stereotaxic coordinates were 4.0 mm ventral to bregma, 1.5 mm right lateral, and 0.8 mm posterior. The guide cannula was fixed to two stainless screws fixed to the skull and sealed with a cannula rod. The lentiviral vectors carrying miR‐223‐3p (Lv‐miR‐223‐3p) and negative control (Lv‐NC) were provided by GenePharma Co., Ltd (Shanghai, China). From day 0 to day 6 after surgery, 10 μl of recombinant lentivirus (10^7^ P.F.U./mouse) were injected into mice through intracerebroventricular administration every other day using a microinjection syringe through a PE‐20 catheter. In one experiment, the mice were divided into the sham group (*n* = 6) and CCI‐ION group on postoperative day 0, 3, 7, 10, and 14, *n* = 6 for each time point. In another experiment, the were divided into the sham group, CCI group, CCI + Lv‐miR‐223‐3p group, *n* = 6 for each group.

### 2.4 Von Frey test

Mechanical sensitivity of the whisker pad, the infraorbital nerve receptive field, was measured with a series of Von Frey fiber filament (Stoelting, Wood Dale, IL). Before tests, each mouse was shaved around the whisker pad and handled for 3 days (once a day for 30 min). One experimenter gently held the mouse in an insulating cotton glove until the animal was calm. A series of calibrated Von Frey filaments ranging from 0.07 to 4 g were lightly applied on the ipsilateral whisker pad. A brisk or active withdrawal of the head from the probing filament was defined as a response. Each filament was tested five times at 5‐s intervals. The withdrawal threshold was defined as the lowest force in grams that produced at least three withdrawal responses in five consecutive applications (Guo et al., 2018; Liang et al., 2007). On the other hand, Von Frey filaments (0.4 g and 2.0 g) were used to stimulate the whisker pad innervated by the ION, and the responses of the mice were recorded. The filaments were applied three times on the ipsilateral whisker pad. The response to each of the filaments is scored as follows: score 0, no response; score 1, detection‐the mice detected and explored the von Frey hair; score 2, head slowly withdrawal reaction; score 3, escape/attack; score 4, asymmetric face grooming‐at least three face wash strokes directed toward the stimulated facial area (see Table 1) (Kernisant et al., 2008; Zhang et al., 2015). All behavioral tests were conducted under blind conditions.

**TABLE 1:** Response scoring system

Response category	Observed response elements				
	Detection	Withdrawal	Escape/attack	Face‐grooming	Score
No response	0	0	0	0	0
Nonaversive response	1	0	0	0	1
Mild aversive response	1	1	0	0	2
Strong aversive response	1	1	1	0	3
Prolonged aversive behavior	1	1	1	1	4

### 2.5 Assessment of glutamate in the cerebrospinal fluid

The mice were placed in a prone position, and the neck was bent at a 45‐degree angle. The occipital bone to the atlas muscle was cut with iris scissors to expose the white dura, and it was punctured at 2 mm between the occipital bone and the atlas cone with a needle. A micropipette was used to extract 2.5 μl of cerebrospinal fluid (CSF). The collected CSF was centrifuged at 250 g for 10 min and was frozen at −80°C. The glutamate level was determined using a Waters high‐performance liquid chromatography (HPLC) system (Waters Breeze 1525) equipped with a UV–Visible detector (Waters 2489), an autosampler (Waters 2707), and an isocratic pump (Waters 1515). Trigeminal ganglions (TGs) from the unilateral surface were dissected immediately and frozen at −80°C for further assays.

### 2.6 RT‐qPCR

Total RNA from TGs was obtained using TRIzol reagent (Invitrogen, USA). A mirPremier microRNA Isolation Kit (Sigma‐Aldrich, USA) was used to extract miRNA. Reverse transcription was performed using a M‐MLV Reverse Transcriptase kit (Invitrogen) and PrimeScript miRNA cDNA Synthesis kit (TaKaRa, Japan). The expression of miR‐223‐3p and MKNK2 was determined using SYBR Green Supermix (Bio‐Rad Laboratories, USA) in an Applied Biosystems 7300 real‐time PCR system. The 2^−ΔΔCt^ method was used for quantification (Livak & Schmittgen, 2001). GAPDH and U6 snRNA acted as internal controls. The primer sequences are shown in Table 2.

**TABLE 2:** Primer sequences for RT‐qPCR

Gene	Primer sequence
miR‐223‐3p	F: 5′‐AGCTGGTGTTGTGAATCAGGCCG‐3′
miR‐223‐3p	R: 5′‐TGGTGTCGTGGAGTCG‐3′
MKNK2	F: 5′‐GTTCGAAGATGTCTATCAGC‐3′
MKNK2	R: 5′‐TTCTAGAACATTCCTATGTCCC‐3′
GAPDH	F: 5′‐GGTTGTCTCCTGCGACTTCA‐3′
GAPDH	R: 5′‐CCCTAGGCCCCTCCTGTTAT‐3′
U6	F: 5′‐CTCGCTTCGGCAGCACA‐3′
U6	R: 5′‐AACGCTTCACGAATTTGCGT‐3′

### 2.7 Western blotting

The TGs were homogenized in RIPA lysis buffer in the ice bath. After centrifugation, the collected supernatant was used for Western blotting. The protein samples (60 μg) were separated by 12% SDS‐PAGE electrophoresis and subsequently transferred to PVDF membranes (Bio‐Rad). Next, the membranes were blocked in a vertical shaker with 5% nonfat dry milk for 1 h, and then incubated in primary antibodies including 1L‐1β (ab91606, 1:5000, Abcam, UK), TNF‐α (ab183218, 1:1000), Erk1/2 (ab184699, 1:10,000), p‐Erk1/2 (ab278538, 1:1000), p38 (ab170099, 1:1000), p‐p38 (ab195049, 1:1000), JNK (ab179461, 1:1000), p‐JNK (ab124956, 1:1000), β‐actin (#4970, 1:1000, Cell Signaling Technology), and MKNK2 (SAB2101483, 1:1000, Merck Millipore). After incubation at room temperature for 30 min, they were put in a 4°C refrigerator and maintained overnight. The next day, the membranes were reheated, washed, and incubated with corresponding second antibodies for 1.5 h. The signal bands were visualized with chemiluminescence reagents (PerkinElmer) and analyzed with Quantity Ones software version 4.5 (BioRad).

### 2.8 Luciferase reporter assay

The MKNK2 3′‐UTR containing either the seed sequence of wild type or mutant miR‐223‐3p binding site was inserted into the pmirGLO vector (Promega, USA). HEK293T cells (purchased from ATCC, USA) were seeded at 1 × 10^6^ cells/well in 24‐well plates were transfected with the pmirGLO‐MKNK2‐Wt/Mut and miR‐223‐3p mimics or scrambled miRNA (synthesized by GenePharma) using Lipofectamine 2000 (Invitrogen). After 48 h, the cells were lysed for measurement with a luciferase reporter assay kit (Promega).

### 2.9 Statistical analysis

Experimental data were analyzed with GraphPad Prism 7 software and are expressed as mean ± SD from at least three independent tests. Differences between groups were compared unpaired t test and one‐way or two‐way ANOVA. Pearson correlation coefficient was applied to investigate the relationship between miR‐223‐3p and MKNK2 levels in TGs. Values of *p* < .05 were statistically significant.

## 3 RESULTS

### 3.1 The miR‑223‐3p level is reduced in a mouse model of CCI‑ION

**FIGURE 1:**
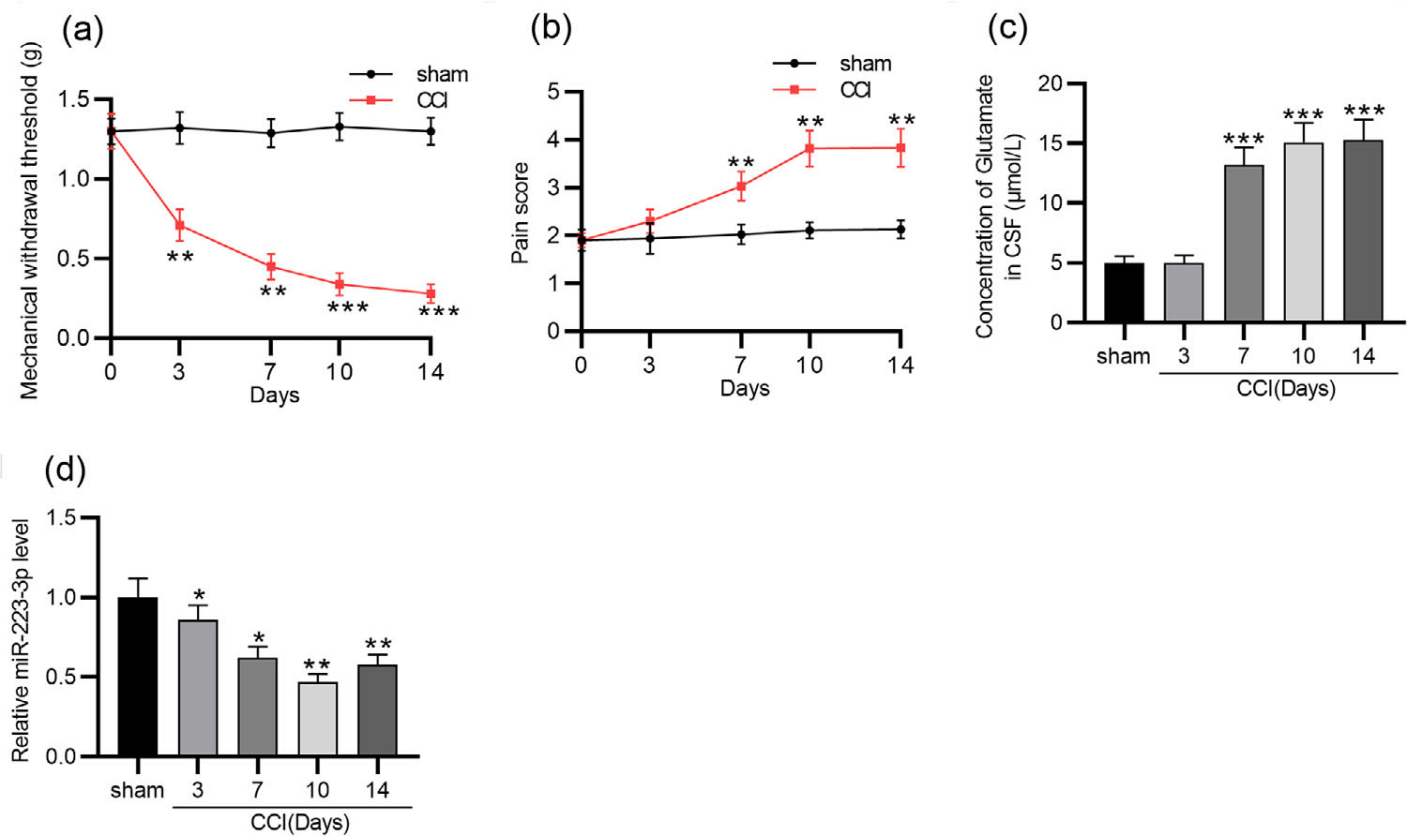
The miR‑223‐3p level in a mouse model of CCI‑ION. (a) Mechanical withdrawal threshold in sham and CCI‐ION mice on postoperative day 0, 3, 7, 10, and 14. (b) Neuropathic pain score in sham and CCI‐ION mice on postoperative day 0, 3, 7, 10, and 14. (c) The concentrations of glutamate in the CSF of sham and CCI‐ION mice at the different time points. (d) RT‐qPCR analysis of miR‐223‐3p expression in TGs of CCI‐ION mice at the different time points. *n* = 6 for each time point. ^*^ *p* < .05, ^**^ *p* < .01, ^***^ *p* < .001

First, mechanical sensitivity was evaluated by Von Frey filament and was comparable between sham group and CCI‐ION‐treated group at the different time points (Figure 1a). Compared to sham group, the mechanical withdrawal thresholds of the CCI‐ION‐treated group were significantly decreased within 14 days postoperation (*p* = .001 on day 3; *p* = .000 on day 7; *p* = .000 on day 10; *p* = .000 on day 14). Additionally, the response score was increased over time postoperation. An increased score indicated that the mice found the stimulation unpleasant or nociceptive. On sham‐operated mice, the behavioral score remained at presurgery levels for the entire period (*p* = .009 on day 7; *p* = .002 on day 10; *p* = .002 on day 14) (Figure 1b). Glutamate is a key neurotransmitter in the nervous system and its level is associated with neuropathic pain (Fernández‐Montoya et al., 2017). It was shown that the CCI‐ION group had a higher glutamate level than the sham group within 14 days (*p* = .000 on day 7; *p* = .000 on day 10; *p *= .000 on day 14) (Figure 1c). The above findings demonstrated that a TN model was successfully established. Next, miR‐223‐3p expression was measured and it was significantly decreased in TGs in CCI‑ION mice on postoperative day 3, 7, 10, and 14, suggesting a possible association between miR‐223‐3p expression and TN progression (*p* = .009 on day 7; *p* = .002 on day 10; *p* = .005 on day 14) (Figure 1d).

### 3.2 Increased miR‑223‐3p expression attenuates trigeminal neuropathic pain

**FIGURE 2:**
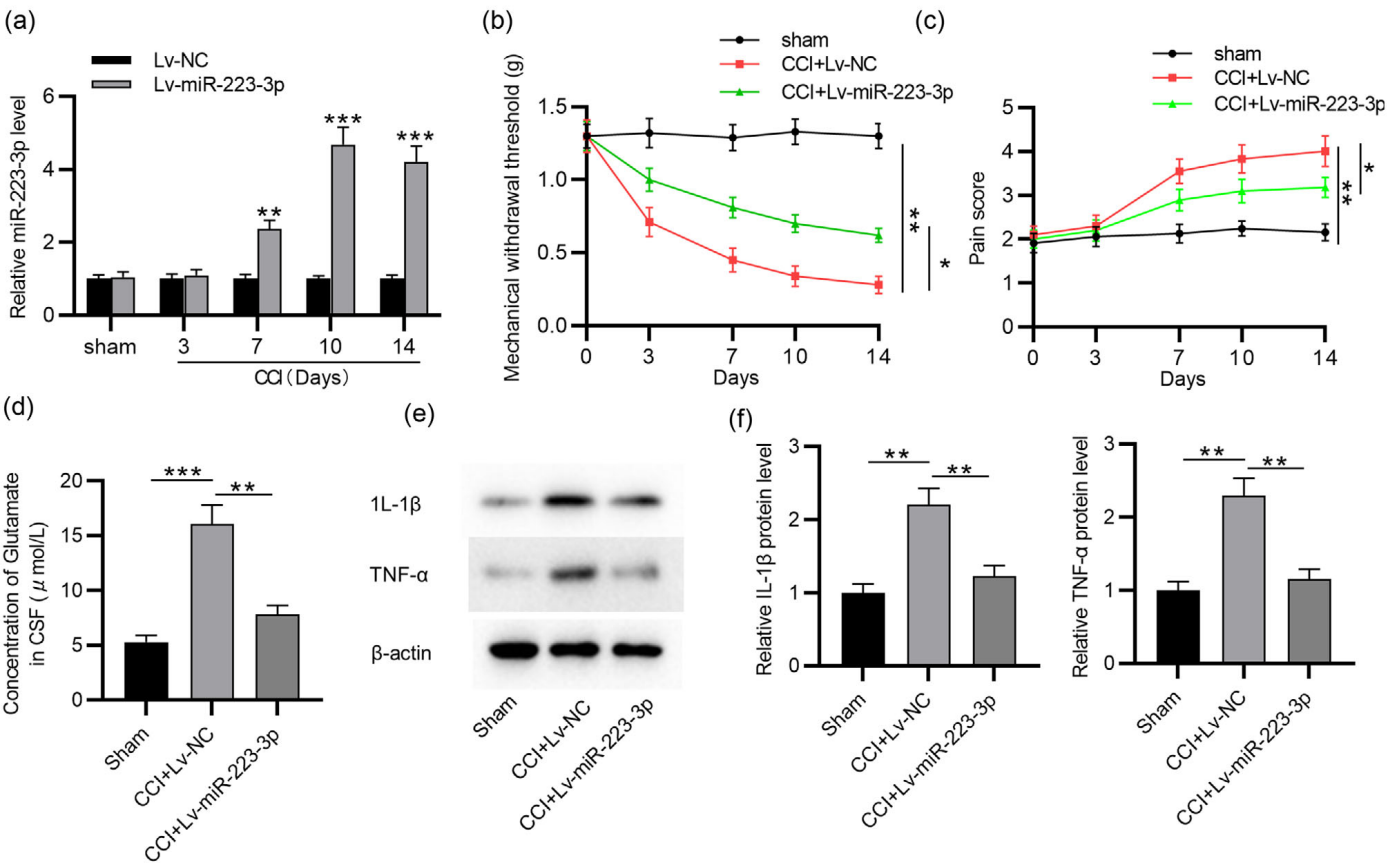
Effects of miR‑223‐3p on trigeminal neuropathic pain. (a) RT‐qPCR analysis of miR‐223‐3p expression in TGs of CCI‐ION mice infected with Lv‐miR‐223‐3p or Lv‐NC on postoperative day 0, 3, 7, 10, and 14. (b,c) Behavioral tests of effects of miR‐223‐3p overexpression on neuropathic pain at the different time points. (d) The concentrations of glutamate in the CSF of CCI‐ION mice infected with Lv‐miR‐223‐3p or Lv‐NC. (e,f) Western blotting analysis of the levels of IL‐1β and TNF‐α in TGs of CCI‐ION mice infected with Lv‐miR‐223‐3p or Lv‐NC. *n* = 6 for each time point. *n* = 6 for each group. ^*^ *p* < .05, ^**^ *p* < .01, ^***^ *p* < .001

MiR‐223‐3p was experimentally overexpressed in mice via intracerebroventricular injection of Lv‐miR‐223‐3p to test the role of miR‐223‐3p in TN. RT‐qPCR showed that miR‐223‐3p was successfully upregulated in TGs of CCI‐ION mice with Lv‐miR‐223‐3p injection (*p* = .001 on day 7; *p* = .000 on day 10; *p* = .000 on day 14) (Figure 2a). Behavior tests revealed that miR‐223‐3p overexpression alleviated ipsilateral mechanical allodynia within 14 days after surgery (*p* = .001) (Figure 2b). The response score in CCI‐ION mice was also decreased after Lv‐miR‐223‐3p injection (*p* = .026) (Figure 2c). Furthermore, glutamate content in the CSF was significantly decreased in CCI‐ION mice after miR‐223‐3p overexpression (*p* = .001) (Figure 2d). To further assess the relationship between neuroinflammation and trigeminal neuropathic pain, the expression of proinflammatory cytokines in TGs was determined. We found that CCI‐ION mice showed upregulated expression of IL‐1β and TNF‐α protein in TGs compared to the sham group, while miR‐223‐3p overexpression reversed these results (*p* = .003 for IL‐1β; *p* = .001 for TNF‐α) (Figure 2e,f). Overall, miR‐223‐3p overexpression attenuates trigeminal neuropathic pain in CCI‐ION mice.

### 3.3 MKNK2 is targeted by miR‐223‐3p

**FIGURE 3:**
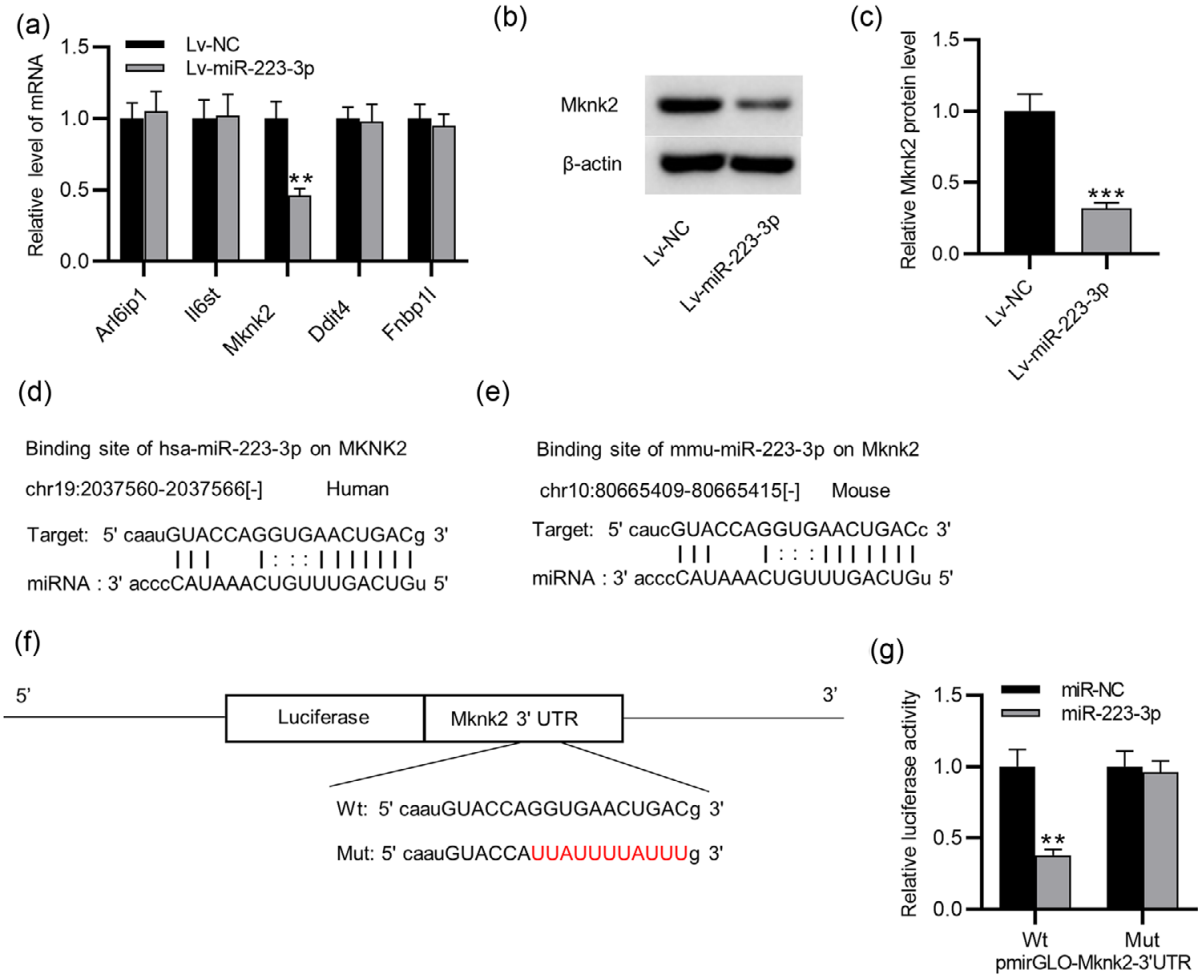
MKNK2 is a targeted by miR‐223‐3p. (a–c) RT‐qPCR and western blotting analysis of MKNK2 expression in TGs of CCI‐ION mice infected with Lv‐miR‐223‐3p or Lv‐NC on day 0, 3, 7, 10, and 14. (d) Binding site of hsa‐miR‐223‐3p in MKNK2. (e) Binding site of mmu‐miR‐223‐3p in MKNK2. (f) Luciferase reporters containing the sequence of MKNK2‐Wt/Mut 3′‐UTR were constructed. (g) HEK293T cells were simultaneously transfected with the luciferase reporter vectors and miR‐223‐3p mimics. The luciferase activity was measured after 48 h. *n* = 6 for each group. ^**^ *p* < .01, ^***^ *p* < .001

The starBase database was examined to identify targets of miR‐223‐3p. Five possible targets having binding site for miR‐223‐3p are shown in Figure 3a (search criteria: AgoExpNum > 7). As RT‐qPCR showed, only MKNK2 was significantly downregulated in TGs of CCI‐ION mice with Lv‐miR‐223‐3p (*p* = .001), and the other four genes had no significant change (Figure 3a). Additionally, the MKNK2 protein expression in TGs of CCI‐ION mice was also reduced when miR‐223‐3p was upregulated (*p* = .000) (Figure 3b,c), demonstrating that miR‐223‐3p negatively modulates MKNK2. We found that the binding site of miR‐223‐3p in MKNK2 is highly conserved in both human and mouse (Figure 3d,e). To examine whether miR‐223‐3p targets the MKNK2 3′‐UTR, luciferase reporters containing the sequence of MKNK2‐Wt/Mut 3′‐UTR were constructed (Figure 3f). HEK293T cells were simultaneously transfected with the luciferase reporter vectors and miR‐223‐3p mimics. The luciferase activity in vectors containing MKNK2‐Wt 3′‐UTR was reduced by miR‐223‐3p mimics (*p* = .001), and the MKNK2‐Mut 3′‐UTR group showed no significant response (Figure 3g). This showed that MKNK2 is targeted by miR‐223‐3p.

### 3.4 MKNK2 is upregulated in CCI‐ION mice

**FIGURE 4:**
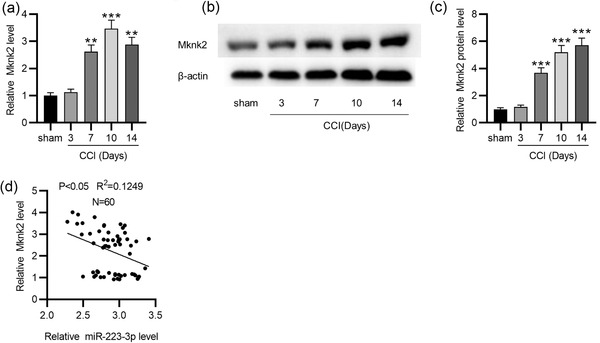
MKNK2 is upregulated in CCI‐ION mice. (a–c) RT‐qPCR and Western blotting analysis of MKNK2 expression in TGs of CCI‐ION mice at the different time points. (d) An association between MKNK2 and miR‐223‐3p levels in TGs of CCI‐ION mice. *n* = 6 for each time point. ^**^ *p* < .01, ^***^ *p* < .001

Next, the MKNK2 level in TGs of CCI‐ION mice was measured using RT‐qPCR and western blotting. In our study, we detected that the MKNK2 mRNA (*p* = .000 on day 7; *p* = .000 on day 10; *p* = .000 on day 14) and protein (*p* = .000 on day 7; *p* = .000 on day 10; *p* = .000 on day 14) expression was markedly higher in TGs in the CCI‐ION group on postoperative day 7, 10, and 14 than in the sham group (Figure 4a–c). Additionally, there was a negative association between MKNK2 and miR‐223‐3p levels in TGs of CCI‐ION mice (Figure 4d).

### 3.5 MiR‑223‐3p inhibits MAPK/ERK signaling in CCI‐ION mice

**FIGURE 5:**
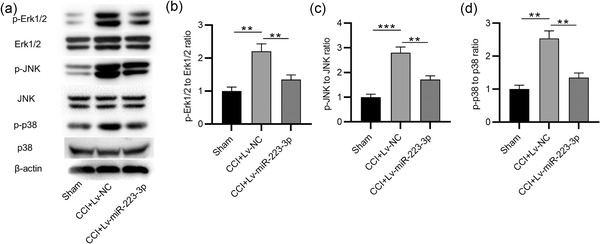
MiR‑223‐3p inhibits MAPK signaling in CCI‐ION mice. (a–d) Western blotting analysis of the phosphorylation levels of ERK1/2, JNK, and p38 protein in TGs of CCI‐ION mice infected with Lv‐miR‐223‐3p or Lv‐NC. *n* = 6 for each group. ^**^ *p* < .01, ^***^ *p* < .001

MKNK2 is a substrate of the MAPK pathway that plays a crucial role in neuropathic pain. We thus examined the MAPK pathway in response to TN development. Western blotting indicated that the phosphorylation levels of ERK1/2, JNK, and p38 protein were significantly upregulated in TGs in the CCI‐ION group, while miR‐223‐3p overexpression reduced the phosphorylation levels of these protein (*p* = .005 for p‐ERK1/2; *p* = .002 for p‐JNK; *p* = .001 for p‐p38), suggesting that miR‐223‐3p inhibits MAPK/ERK signaling in CCI‐ION mice (Figure 5a–d).

## 4 DISCUSSION

A multitude of miRNAs are shown to be differentially expressed in various nervous tissues with pain (Huang et al., 2016; Leinders et al., 2016). However, there are a few studies concerning differential expression of miRNAs in neuropathic pain associated with trigeminal nerve injury. Previous studies revealed that inflammatory pain results in downregulation of miR‐183, 10a, −124a, −29a, −134, −98, −125a‐3p, and −99a in TGs (Bai et al., 2007). MiR‐223 is transcribed from an independent promoter and expressed specifically in the hematopoietic system (Yuan et al., 2018). Dysregulation of miR‐223‐3p has been described in many diseases. Overexpression of miR‐223‐3p protects dissociated cortical neurons from condition media‐mediated degeneration, and introduction of miR‐223‐3p in vivo in mouse retinal ganglion cells protects their axons from degeneration in experimental autoimmune encephalomyelitis (Morquette et al., 2019). MiR‐223‐3p is involved in innate immune responses by regulating myeloid differentiation and granulocyte functions (Aziz, 2016; Haneklaus et al., 2013). MiR‐223‐3p was found to be downregulated in several tumor types and is known to be involved in regulating cell growth and apoptosis (Fang et al., 2017; Wang et al., 2019), highly suggestive of its role in regulating various cellular functions.

Reduced expression of miR‐223 in TGs was detected after facial inflammatory pain induced by complete Freund's adjuvant (Chen et al., 2018). Intriguingly, here, miR‐223‐3p showed the significant downregulated expression in TGs of CCI‐ION mice. Additionally, miR‐223 suppresses the activities of NLRP3 inflammasomes to relieve morphine analgesic tolerance in rats (Xie et al., 2017) and alleviates neuropathic pain in a mice model of chronic sciatic nerve injury (Zhu et al., 2021). In this study, aggravated allodynia was detected in mice after CCI‐ION surgery. We administered Lv‐miR‐223‐3p in experimental models through intracerebroventricular catheter, contributing to the overexpression of miR‐223‐3p. Our results revealed that Lv‐miR‐223‐3p administration significantly attenuated facial pain in CCI‐ION mice, suggesting that miR‐223‐3p may inhibit TN development. TNF‐α and IL‐1β are proinflammatory cytokines that can increase the excitability of neurons and accelerate the development of pain. Promoting the release of anti‐inflammatory cytokines and blocking the cytokine cascade reaction are crucial to control the pain (Q. Y. Li et al., 2017). MiR‐223‐3p has been reported to inhibit the activity of inflammasome and participate in modulating inflammation (Ding et al., 2018; H. C. Dong et al., 2019). We found that Lv‐miR‐223‐3p administration significantly decreased the expression of IL‐1β and TNF‐α protein in TGs of CCI‐ION mice. Similar to this, Zou et al. (2021) found that miR‐223‐3p overexpression inhibits inflammation and nerve cell injury in postherpetic neuralgia. Overall, miR‐223‐3p may play an antalgic role in TN. However, only one sex of the mice (male) was used in this study. Sex‐specific susceptibility to pain conditions as well as sex differences in pain sensitivity, pain tolerance, and analgesic efficacy are increasingly recognized in the literature and have thus prompted scientists to seek mechanistic explanations (Midavaine et al., 2021). Therefore, it is necessary to investigated potential sex‐based differences in the process of TN in the future work.

We further found that the MKNK2 mRNA and protein expression was reduced in TGs of the TN group, suggesting that MKNK2 is negatively related to miR‐223‐3p expression. The targeted relationship between MKNK2 and miR‐223‐3p was demonstrated in this study. Translational modulation of gene expression is a key event in neuronal plasticity, displaying dispensable roles in pathophysiology of pain (Uttam et al., 2018). A study demonstrated that MKNK2 is involved in mechanisms of nociceptor plasticity and chronic pain development (Moy et al., 2017). And MKNK inhibition was shown to reduce sensitization of dorsal root ganglion nociceptors (Jeevakumar et al., 2020). The kinase MKNK2 was found by its interaction with the mitogen‐activated protein kinases (MAPKs), p38‐MAPK, and extracellular signal‐regulated kinase (ERK) (Waskiewicz et al., 1997). MAPK is a conserved kinase response signaling containing ERK1/2, ERK5, and p38‐MAPK (Schreiber et al., 2006). It has been shown that MAPK activation in the spinal cord or primary afferent nerve promotes nociceptor hyperexcitability and induces changes in neuronal plasticity, which is associated with neuropathic and inflammatory pain (Yu et al., 2016). As reported, scorpion analgesic peptide N58A exerts significant analgesic effects on CCI‐ION rat models possibly by decreasing the phosphorylation levels of MAPK signaling protein (C. L. Li et al., 2021). Additionally, miR‐125a‐3p attenuates the maintenance of orofacial inflammatory pain through negatively regulating p38 MAPK in rat trigeminal ganglions (Y. Dong et al., 2014). Therefore, inhibiting activation of MAPK pathway may be a promising tool in treating trigeminal neuropathic pain. Similar to previous findings, we showed that miR‐223‐3p overexpression reduced the phosphorylation levels of ERK1/2, JNK, and p38 protein in TGs of CCI‐ION mice, suggesting that the analgesic effects of miR‐223‐3p may be achieved by inhibiting MAPK pathway.

In conclusion, this study was the first to elucidate the downregulation of miR‐223‐3p expression in CCI‐ION models of TN. Moreover, miR‐223‐3p overexpression was demonstrated to mitigate trigeminal neuropathic pain and inhibit neuroinflammation, and these effects may relate to the targeted relationship with MKNK2 and inhibition of the MAPK pathway. In the future work, we will establish the other experimental models such as the ligation of infraorbital nerve to investigate the antalgic role of miR‐223‐3p. Additionally, more upstream and downstream molecular mechanisms involved in miR‐223‐3p need to be investigated for deep understanding of the physiology of TN.

## CONFLICT OF INTEREST

The authors declare that they have no competing interests.

## Data Availability

The datasets used or analyzed during the current study are available from the corresponding author on reasonable request.
